# Regulation of NFκB Signalling by Ubiquitination: A Potential Therapeutic Target in Head and Neck Squamous Cell Carcinoma?

**DOI:** 10.3390/cancers12102877

**Published:** 2020-10-07

**Authors:** Ethan L. Morgan, Zhong Chen, Carter Van Waes

**Affiliations:** Tumor Biology Section, Head and Neck Surgery Branch, National Institute of Deafness and Other Communication Disorders, NIH, Bethesda, MD 20892, USA; vanwaesc@nidcd.nih.gov

**Keywords:** head and neck cancer, NFκB, ubiquitin, E3 ligases, deubiquitinases

## Abstract

**Simple Summary:**

Head and neck cancer is the sixth most common cancer worldwide and typically caused by smoking and alcohol consumption, or infection with Human Papillomavirus (HPV). Currently, treatment options include surgery, radiotherapy, and/or chemotherapy. However, whilst the survival rate in HPV+ cancer patients is better than those without HPV, survival rates have not improved greatly in recent years, and recurrence rates are high. Ubiquitination is a critical regulatory mechanism that can promote the activation or termination of signal cascades, such as the NFκB pathway, which plays an important role in the pathogenesis and therapeutic resistance of head and neck cancer. In this review, we discuss how NFκB signalling is regulated by ubiquitination and how the ubiquitin pathway is deregulated in head and neck cancer, highlighting how the this pathway may be targeted to inhibit NFκB signalling.

**Abstract:**

Head and neck squamous cell carcinoma (HNSCC) is the sixth most common cancer worldwide, with over 600,000 cases per year. The primary causes for HNSCC include smoking and alcohol consumption, with an increasing number of cases attributed to infection with Human Papillomavirus (HPV). The treatment options for HNSCC currently include surgery, radiotherapy, and/or platinum-based chemotherapeutics. Cetuximab (targeting EGFR) and Pembrolizumab (targeting PD-1) have been approved for advanced stage, recurrent, and/or metastatic HNSCC. Despite these advances, whilst HPV+ HNSCC has a 3-year overall survival (OS) rate of around 80%, the 3-year OS for HPV− HNSCC is still around 55%. Aberrant signal activation of transcription factor NFκB plays an important role in the pathogenesis and therapeutic resistance of HNSCC. As an important mediator of inflammatory signalling and the immune response to pathogens, the NFκB pathway is tightly regulated to prevent chronic inflammation, a key driver of tumorigenesis. Here, we discuss how NFκB signalling is regulated by the ubiquitin pathway and how this pathway is deregulated in HNSCC. Finally, we discuss the current strategies available to target the ubiquitin pathway and how this may offer a potential therapeutic benefit in HNSCC.

## 1. Introduction

Head and neck squamous cell carcinoma (HNSCC) is the sixth most common cancer worldwide, with over 600,000 cases and around 300,000 deaths per year [1]. Current therapies include surgery, radiotherapy, and standard chemotherapies [2]. However, targeted therapies have been approved for use in the advanced stage, recurrent, and/or metastatic HNSCC, including anti-epidermal growth factor receptor (EGFR) (cetuximab) and anti-programmed cell death protein (PD-1) therapy (pembrolizumab) [3,4,5]. Despite these advances, whilst HPV+ HNSCC has a 3-year overall survival (OS) rate of around 80%, the 3-year OS for HPV− HNSCC is still around 55% [1]. While the early stage disease has survival rates of 70–90%, the locally advanced disease can have survival rates <50% [1].

The recent increase in survival rate is largely due to an epidemiological shift in the causes of HNSCC. In low- and middle-income countries, the primary known risk factors for most HNSCC cases (>90%) remain alcohol and tobacco use [6]. However, in high-income countries, such as the USA and Western Europe, over 70% of cases involve infection with human papillomavirus (HPV) [1,7]. HPV is an important carcinogenic factor, accounting for around 5% of all cancers worldwide [8]. The most common cancer associated with HPV infection is cervical cancer, which is primarily caused by the high-risk HPV types 16 and 18 (70%) [9]; in contrast, HPV+ HNSCC is almost always due to HPV16 infection (over 90%) [1]. Interestingly, the incidence of HPV+ oropharyngeal cancers is higher in current smokers than never smokers, suggesting that smoking may also play a role in the clinical outcome of these patients [10].

The differences between these cancers manifest in several characteristics (Table 1). HPV− cancers are decreasing, occur in older adults, and localized in any region of the head and neck; in contrast, HPV+ HNSCC cases are increasing, mostly occurs in younger adults and is localized primarily in the oropharynx and the base of the tongue (BOT) [1,2]. In clinical terms, HPV− HNSCC are primarily well differentiated with a generally keratinized histology, whereas HPV+ HNSCC tumors are usually poorly differentiated or have basaloid histology [11]. HPV+ HNSCC also have a better prognosis, respond better to chemoradiation therapy, and have higher survival rates than HPV− HNSCC [1]. Thus, while most HPV+ HNSCC patients are diagnosed at a clinically advanced stage with regional lymph node metastases, after treatment, they are less likely to develop local-regional recurrence, secondary malignancies, or distant metastases. Thus, it is clear that HNSCC cases can be separated into (at least) two distinct subtypes, dependent on HPV status, that result in different clinical outcomes.

As one of the hallmarks of cancer [12], the role of inflammation in tumor initiation and progression has become an important area of investigation. Inflammatory conditions can induce signalling and genetic damage to initiate or promote tumorigenesis; furthermore, genetic and epigenetic changes, as well as the HPV oncogenes, can generate an inflammatory microenvironment that further supports tumor progression [6,13]. Many inflammatory cytokines and signalling pathways are implicated in the pathogenesis of HNSCC [14,15]; the expression of these inflammatory mediators and intrinsic resistance of tumor cells can be driven by the oncogenic insults known to contribute to the development of HNSCC [14,16,17].

An important driver and inducer of inflammatory mediators, which promotes cell survival and therapeutic resistance, is the transcription factor nuclear factor kappa-light-chain-enhancer of activated B cells (NFκB) [18]. In HNSCC, NFκB is often aberrantly activated and is a common mediator of the effects of numerous pro-inflammatory cytokines (TNFα and IL-1), as well as other signalling pathways targeted by oncogenic mutations, genetic copy number alterations (CNA), or viral oncogenes [14,19,20]. NFκB drives the expression of a repertoire of genes that promote malignant progression, including cell proliferation and survival, therapeutic resistance, and pro-inflammatory factors [21,22].

An important process in the regulation of signalling pathways is protein ubiquitination, which plays a pivotal role in the activation and termination of many signalling pathways, including the NFκB pathway [23]. Thus, defects in ubiquitination can result in aberrant NFκB activation, or the failure to terminate NFκB signalling.

In this review, we will discuss how the ubiquitin system regulates NFκB signalling and how this may contribute to the aberrant NFκB activity observed in HNSCC. Additionally, we will summarize the current methods of modulating the ubiquitin system that could potentially be used to inhibit NFκB signalling in HNSCC.

## 2. The NFκB Signalling Pathway

The difference in clinical response of HPV+ and HPV− HNSCC is not fully understood; however, recent developments in the genomic and transcriptomic analysis of primary HNSCC cases have provided insight into the difference in clinical response [14,17]. Subsequent studies have examined the alterations in specific pathways and how these relate to the pathogenesis of HNSCC and have highlighted a critical role for aberrant NFκB activity.

The NFκB family of transcription factors are homo- or heterodimers that regulate a large number of genes involved in inflammation, immunity, and cell survival [24]. In humans, the NFκB family has five known members: p105/p50 (NFκB1), p100/p52 (NFκB2), p65 (RELA), c-REL, and RELB [24]. Each family member contains an N-terminal Rel homology domain (RHD), regulating their dimerization, DNA binding ability, and nuclear localization. The RHD also binds to the inhibitory of κB proteins (IκBs), critical regulators of NFκB, which sequester it in the cytoplasm in unstimulated cells [24]. p50 and p52 do not contain their own transactivation domains (TAD); they are generated from the precursor proteins, p105 and p100, respectively, following phosphorylation, ubiquitination, and proteasomal degradation of a C-terminal Inhibitor-kappaB kinase (IκB)-like domain [25,26]. Homodimers of p50 or p52 cannot activate gene transcription without additional co-factors and therefore function as gene repressors [27]. p50 and p52 can also bind to a TAD-containing family member to generate a functional NFκB heterodimer. p65, c-REL and RELB contain a TAD capable of activating gene expression. The TAD of RELs contains sites for post-translational phosphorylation and/or acetylation, which enhances transactivation and duration of DNA binding [28].

The IκB family consists of several ankyrin repeat-containing proteins, including IκBα, IκBβ, IκBɛ, IκB-δ (IκBNS), IκB-ζ, and BCL-IκBα, IκBβ and IκBɛ inhibit DNA binding and enhance export and sequestration of NFκB in the cytoplasm, whereas IκB-δ, IκB-ζ, and Bcl-3 function in the nucleus as co-activators of NFκB [29]. Whereas the majority of IκBs serve as inhibitors of NFκB, the IκBζ, and Bcl-3 isoforms instead potentiate NFκB transactivation in the nucleus [30].

### 2.1. Canonical NFκB Signalling

The mechanisms of activation and function of the canonical (classical) and non-canonical (alternative) NFκB pathways are often distinct; however, studies have revealed that cross-activation of these pathways occurs at several levels via kinase function, processing of p100/p105 precursors, and transcription [31]. The canonical NFκB pathway plays an important role in biological processes such as innate and adaptive immunity and cell survival. A variety of stimuli activate canonical NFκB signalling, which converges upon a trimeric IκB kinase (IKK) complex, consisting of catalytic subunits IKKα and IKKβ and the regulatory subunit NFκB essential modifier (NEMO, also known as IKKγ). IKKβ and NEMO serve essential and non-redundant roles in the activation of canonical NFκB signalling, whereas IKKa regulates both canonical and non-canonical pathways [31]. IKKα and IKKβ can phosphorylate IκB proteins at two N-terminal serine residues, triggering their ubiquitination and proteasomal degradation, thus allowing NFκB to enter the nucleus and activate stimulus-specific gene programs.

**Figure 1:**
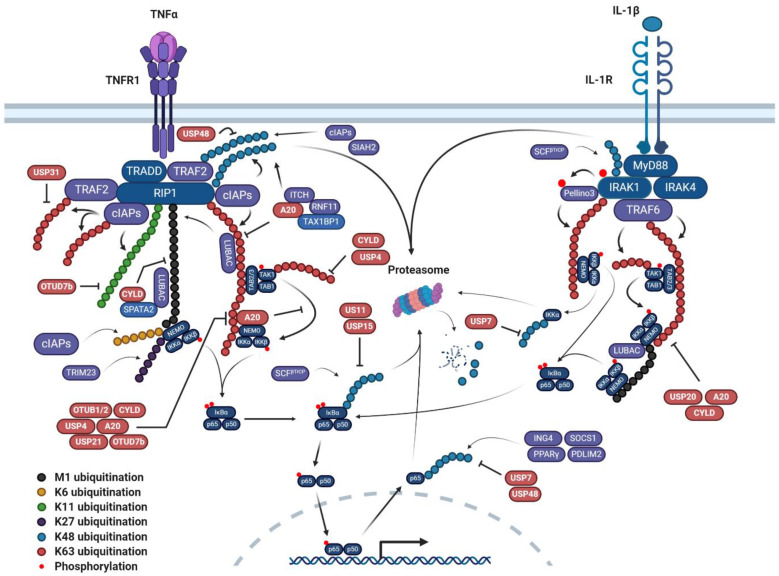
The canonical NFκB signalling pathway induced by TNF-α and IL-1β and the role of ubiquitination. In TNFα signalling, binding of TNFα to its receptor TNFR1 leads to the recruitment of TRADD, TRAF2, RIP1 and cIAPs. This leads to K63-polyubiquitination on TRAF2, cIAP1/2 and RIP1, providing a binding platform for the TAK1 complex via TABIn IL-1β signalling, binding of IL-1β to its receptor IL-1R allows binding of the adapter protein MyD88 to the receptor TIR domain, resulting in the recruitment of the kinases Interleukin 1 Receptor Associated Kinase (IRAK) 1 and IRAKIRAK1 and 4 can then dissociate from the receptor and interact with the E3 ligase TRAF6, leading to the formation of K63-polyubiquitin chains on TRAF6 and IRAK1, serving as a binding platform for the TAK1 complex via TABIrrespective of the stimuli, LUBAC binding to K63-polyubiquitin chains on cIAPs stabilize the complex and induce M1-polyubiquitin chains on RIP1 and the recruitment of IKK complex via NEMO. The IKK complex is then activated by TAK1, resulting in the phosphorylation of IκBα and its subsequent proteasomal degradation. This allows nuclear translocation of the NFκB subunits and NFκB activation. The other proteins and ubiquitin linkages depicted in the figure are discussed in the text. For clarity, E2 ligases and some E3 ligases are not shown in the figure but are discussed in the text. Dark blue, core NFκB pathway components; Light blue, adapter proteins; Purple, E3 ligases; Red, Deubiquitinases.

Canonical NFκB pathway is exemplified by activation via proinflammatory cytokines such as Tumor Necrosis Factor α (TNFα) or Interleukin-1β (IL-1β) (Figure 1). In TNFα mediated signalling, TNFα binding to the TNF receptor (TNFR1) initiates recruitment of a complex consisting of Tumor necrosis factor receptor type 1-associated DEATH domain (TRADD), TNF-receptor-associated factor 2 (TRAF2), receptor-interacting serine/threonine-protein kinase 1 (RIP1), and cellular Inhibitor of Apoptosis 1/2 (cIAP1/2). This leads to the K63-polyubiquitination of RIP1 by the heterodimer UBC13-UBE2V1, an E2 protein complex, and cIAP1/2, which are E3 ligases [32].

The resulting poly-ubiquitin chains form a scaffold for the recruitment of TAB2 and NEMO and activation of the transforming growth factor β-activated kinase 1 (TAK1) and IKK complexes, respectively. Subsequently, TAK1 induces the phosphorylation of IKKβ, which subsequently induces IκBα phosphorylation, proteasomal degradation, and NFκB nuclear translocation and activation [33,34].

In IL-1 signalling, the ligand-bound IL-1 receptor (IL-1R) recruits the adapter protein MyD88, resulting in the recruitment of the kinases Interleukin 1 Receptor Associated Kinase (IRAK) 1 and IRAK4 [35]. IRAK1/ 4 can then dissociate from the receptor and interact with the E3 ligase TRAF6, leading to the formation of K63-polyubiquitin chains on TRAF6 and IRAK1, serving as a binding platform for the TAK1 complex via TAB2 [36]. As with TNFα signalling, TAK1 then activates the IKK complex [33].

### 2.2. Non-Canonical NFκB Signalling

**Figure 2:**
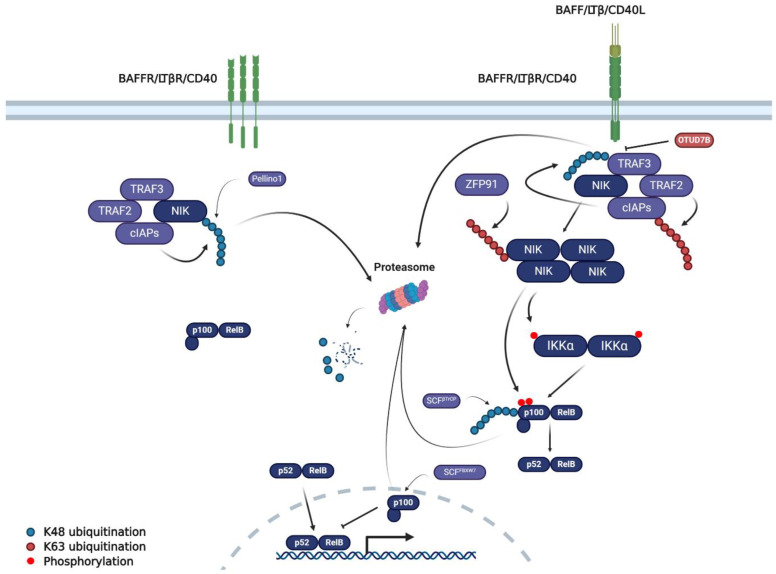
The non-canonical NFκB signalling pathway and the role of ubiquitination. In the absence of ligand (left), the TRAF2/TRAF3/cIAP complex facilitates the proteasomal degradation of NIK. This results in the sequestration of RelB in the cytoplasm by p100, the precursor of the NFκB protein pFollowing ligand: receptor engagement, TRAF3 is recruited to the receptor and is degraded by the combined functions of TRAF2 and cIAP1/The loss of TRAF3 allows the accumulation of NIK, leading to the phosphorylation and activation of IKKα. IKKα then facilitates the partial degradation of p100, allowing the activated p52/RelA heterodimer to translocate into the nucleus. The other proteins and ubiquitin linkages depicted in the figure are discussed in the text. For simplification, E2 ligases are not shown in the figure. Dark blue, core NFκB pathway components; Purple, E3 ligases; Red, Deubiquitinases.

While the canonical NFκB pathway can be activated by several inflammatory cytokines and other stimuli, the non-canonical, or alternative, NFκB pathway is activated by a different set of cytokine/receptor pairs in the TNFR superfamily (Figure 2). These typically do not contain death domains and include the BAFF receptor (BAFFR), CD40, and lymphotoxin B receptor (LTβR), activated by BAFF, CD40L, and LTβ, respectively [37]. Non-canonical NFκB signalling is driven by NFκB-inducing kinase (NIK); in unstimulated cells, NIK expression is low due to its persistent ubiquitination and proteasomal degradation via the TRAF3 E3 ligase in a complex containing TRAF2, TRAF3, and cIAP1/2 [38].

Upon ligand binding, TRAF3 is polyubiquitinated and degraded by the TRAF2/TRAF3/cIAP complex, allowing the cytoplasmic accumulation of NIK. This allows NIK to induce the phosphorylation and activation of IKKα, which in turn phosphorylates p100, promoting its partial proteasomal degradation. This liberates the p52/RELB dimer which can then translocate into the nucleus to drive gene expression [37].

### 2.3. Ubiquitin-Mediated Regulation of NFκB Signalling Pathway 

**Figure 3:**
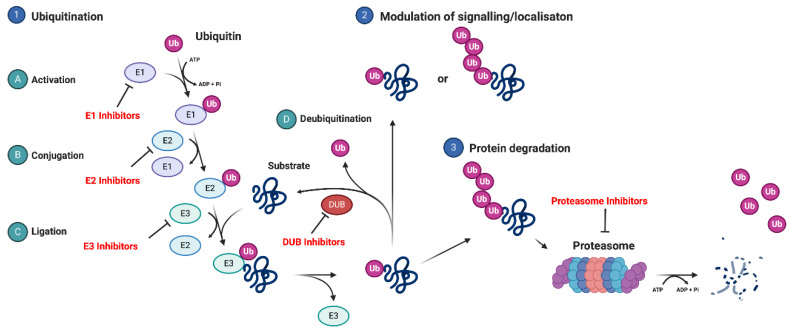
The ubiquitin enzymatic cascade and the ubiquitin-proteasome system (USP). (1) A diagram of the ubiquitin enzymatic cascade. Ubiquitin is added to protein substrates via the E1-E2-E3 enzymatic cascade; (A) E1 activating enzymes, which catalyze the addition of ubiquitin to E1 enzymes is ATP-dependent [39]; (B) E2 conjugating enzymes; (C) E3 ligases, which catalyze the addition of ubiquitin to protein substrates and (D) deubiquitinases, which catalyze the removal of ubiquitin from protein substrates. The addition of ubiquitin to protein substrates can have serval consequences, including (2) the modulation of signal transduction or protein recruitment and localization or (3) proteasomal degradation, which requires ATP. Highlighted in red are components of the ubiquitin system that have been targeted with small molecule inhibitors and are discussed in this review.

Ubiquitination plays an essential role in the regulation of NFκB [23]. Ubiquitin is a small, highly conserved, ubiquitously expressed protein found in all eukaryotic species, from yeast to humans [39]. Ubiquitin regulates a wide range of cellular functions, including protein degradation and the modulation of signal transduction, which is achieved through the conjugation of ubiquitin to protein substrates through a stepwise, enzymatic cascade [40,41]. This process requires three enzyme classes: E1 ubiquitin-activating enzymes, E2 ubiquitin-conjugating enzymes, and E3 ubiquitin ligases (Figure 3). The human genome encodes for eight E1 proteins (only two E1 proteins are involved in ubiquitination), around 50 E2 proteins, and over 700 E3 proteins, demonstrating the complexity of the ubiquitin system [41]. In addition, ubiquitin can be removed by deubiquitinating enzymes (DUBs), of which there has been around 100 characterized in 7 distinct families [42].

Ubiquitin contains 76 amino acids, including seven lysine residues—at amino acid position 6 (K6), K11, K27, K33, K48, and K63; the C-terminus of ubiquitin can be conjugated to any of these seven lysines on another ubiquitin, forming polyubiquitin chains of different linkages [43] Additionally, ubiquitin can be directly attached to the N-terminal methionine of another ubiquitin to form linear polyubiquitin chains [43].

On a structural basis, three types of ubiquitin linkages have been identified: monoubiquitination, polyubiquitination, and branched ubiquitination [40]. Each type of ubiquitination, and upon which lysine ubiquitin conjugation occurs, results in different biological outcomes, demonstrating the complexity of the ubiquitin system. Monoubiquitination is involved in the regulation of DNA damage repair; for example, RNF2-mediated monoubiquitination of γH2AX is required for the initiation of the DNA damage response (DDR) via the recruitment of Ataxia-Telangiectasia Mutated (ATM) and Mediator of DNA damage checkpoint protein 1 (MDC1) [44]. K48-polyubiquitination is most commonly associated with marking its protein substrate for proteasomal degradation [45]; however, it also has a proteasome-independent function [46]. K63-polyubiquitination is involved in creating ligand-receptor mediated scaffolds for signalling proteins, exemplified by its role in the activation of NFκB signalling [32]. K6-polyubiquitination has mostly been associated with the response to DNA damage; however, it may also have an important role in endoplasmic reticulum (ER) homeostasis and mitophagy [47]. K27, K29, and K33-polyubiquitination are the least understood ubiquitin linkages. K27-polyubiquitination is associated with the regulation of the DDR [48], whereas K29-polyubiquitination is associated with the proteasomal function [49]. K33-polyubiquitination plays an important role in anterograde trafficking [50].

The most well studied polyubiquitin chain is K48-polyubiquitination, which promotes the proteasomal degradation of protein substrates [45]. K11-polyubiquitination can also promote proteasomal degradation [51]; in addition, K11-polyubiquitination plays an important role in NFκB signalling [52]. Finally, the M1 linear-polyubiquitination has a primary role in promoting NFκB signalling [53].

Branched ubiquitin chains are poorly understood, likely due to detection difficulties [54]. Branched K48-/K11 and K48-/K29-polyubiquitination are strong degradation signals [54]. Branched K63-/K48-polyubiquitination enhances NFκB signalling by preventing K63-polyubiquitin deubiquitination [55]; however, K63-polyubiquitination also can promote the formation of branched K63-/K48- polyubiquitination, promoting proteasomal degradation [56].

Ubiquitination is a reversible post-translational modification and can be removed by deubiquitinases (DUBs). Over 100 DUBs have currently been characterized in seven distinct families [42]: ubiquitin C-terminal hydrolases (UCHs), ubiquitin-specific proteases (USPs), ovarian tumor proteases (OTUs), the Machado-Josephin domain superfamily (MJD), the MINDY family, and the ZUFSP family members function as cysteine proteases, whereas JAB1/MPN/MOV34 metalloenzymes (JAMMs) are zinc-dependent metalloproteases [42]. These enzymes have different specificities; some cleave only one kind of ubiquitin linkage (e.g., OTULIN; M1 linkages) or multiple ubiquitin linkages (e.g., CYLD; primarily M1 and K63 linkages) [57].

#### 2.3.1. Canonical NFκB Signalling

At the receptor level, an essential step in cytokine mediated NFκB activation is the K63-polyubiquitination of RIP1 and NEMO. Several studies have demonstrated that the E2 proteins UBC13/UEV1A are important regulators of K63-polyubiquitination; these proteins act in concert with cIAP1/2 and TRAF6 to induce K63-polyubiquitination on RIP1 and NEMO, respectively [58]. Additionally, the E3 ligase Parkin can induce RIP1 K63-polyubiquitination (Figure 2; [59]). K63-polyubiquitin chains on RIP1 serve as a binding platform for the regulatory proteins TAB1 and TAB2 through their ubiquitin binding domains (UBDs) [60].

This leads to the recruitment, autophosphorylation and activation of TAK1, and subsequent downstream activation of the IKK complex and IκBα degradation [33]. Furthermore, the K63-polyubiquitination of TAK1 by TRAF2 and TRAF6 is essential for TAK1 activation and downstream signalling [32]. Additionally, NEMO itself can bind to K63-polyubiquitination on RIP1; this brings the IKK complex into close proximity to TAK1, promoting the phosphorylation and activation of the IKK complex [61].

In IL-1β signalling, activation of IRAK1 via autophosphorylation results in the phosphorylation of the E3 ligase Pellino 3; this induces Pellino 3-dependent K63-polyubiquitination of IRAK1, recruitment of NEMO and the downstream activation of NFκB activity [62].

Linear ubiquitination also plays a key role in NFκB activation. M1 linear- polyubiquitination is mediated by the E3 ligase Linear UBiquitin chain Assembly Complex (LUBAC) in concert with the E2 protein UBCH7; LUBAC contains the subunits HOIL-1L, HOIP, and Sharpin [63]. Upon K63-polyubiquitination of RIP1 or TRAF6, LUBAC is recruited and mediates the linear ubiquitination of RIP1 and/or NEMO; these functions are essential for canonical NFκB signalling [53].

In addition to K63- and linear polyubiquitination, components of the NFκB are also extensively modified by atypical polyubiquitin chains. RIP1 is also K11-polyubiquitinated; this is mediated by UBCH5 and cIAP1 [52]. These K11-polyubiquitin chains are TNFα inducible and were bound by NEMO, suggesting they may play an important role in downstream NFκB activation [52]. Furthermore, TNFα can induce UBCH5C/cIAP1 mediated K6-polyubiquitination on NEMO, which mediates TNFα-induced NFκB activation [47]. The E3 ligase tripartite motif protein 23 (TRIM23) can induce K27-polyubiquitin chains in response to viral infection, as this promotes anti-viral NFκB activity [64].

A major step in the activation of the canonical NFκB pathway is the proteasomal degradation of its negative regulator IκBα via the addition of K48-polyubiquitin chains, which occurs after phosphorylation of IκBα at serine 32 and 36 by the IKK complex [34]. IκBα ubiquitination is mediated by a UBC4/5 family member and the E3 ligase Skp1–Cullin1–F-box protein (SCF)^β-TrCP^ (Figure 2; [34]). The degradation of IκBα releases the p65/p50 heterodimer, unveiling their nuclear localization sequences and cleft for DNA binding. Thus, proteasomal degradation of IκBα is essential for the activation of the canonical NFκB pathway. SCF^β-TrCP^ also promotes the degradation of IRAK1 in an IL-1β-dependent manner; this is required for TAK1-mediated NFκB activation [36].

In addition to the activation of NFκB, ubiquitination can also inhibit NFκB signalling. cIAP1 and SIAH2 promote the K48-polyubiquitination of TRAF2, attenuating NFκB activity [65,66]. Further downstream, K48-polyubiquitination of IKKα and β results in their proteasomal degradation, inhibiting NFκB activity [67]. IKKβ K48-polyubiquitination is mediated by the KEAP1-Cul3-RBX1 E3 ligase complex [67]; however, the E3 ligase for IKKα is unclear. The E3 ligase ITCH induces K48-polyubiquitinated of TAK1 [68]. Furthermore, the E3 ligase TRAF7 induces K29-polyubiquitination of NEMO and p65, facilitating the lysosomal degradation of NEMO and p65, inhibiting NFκB activity [69]. Additionally, the NFκB protein p65 is K48-polyubiquitinated by several E3 ligases, including inhibitor of growth protein 4 (ING4) [70], suppressor of cytokine signalling 1 (SOCS1) [71], Peroxisome proliferator-activated receptor γ (*PPARγ)* [72], HERC (82) and PDZ- and LIM-domain containing protein 2 *(PDLIM2)* [73].

The removal of ubiquitin by deubiquitinases (DUBs) also plays a key role in the NFκB pathway. Well characterized DUBs involved in NFκB signalling are A20 (TNFα-Induced Protein 3, TNFAIP3, a member of the OTU family, and CYLD, a member of the USP family [74,75]. Unusually, A20 can also function as a deubiquitinase and an E3 ligase. A20 regulates NFκB signalling via several mechanisms. A20 can remove K63-polyubiquitin chains from RIP1; TNFα stimulation activates NFκB and induces A20 expression as part of a negative feedback loop [76]. IKKα mediated phosphorylation of the adapter protein TAX1BP1 leads to the formation of the A20 ubiquitin-editing complex containing TAX1BP1, ITCH, and RNFThis functions as a deubiquitinase complex to remove K63-polyubiquitin chains from RIP1 [77]. A20 also induces K48-polyubiquitination of RIP1 and UBC13, promoting their proteasomal degradation [78]. Furthermore, A20 inhibits the interaction between the UBC13 and TRAF2/6 and cIAP1/2, inhibiting K63-polyubiquitin chain formation [79]. Additionally, the binding of A20 to K63-polyubiquitin chains of RIP1 can inhibit TAK1-mediated phosphorylation of IKKβ [80]. Finally, A20 binds to linear-polyubiquitin chains, preventing the association between LUBAC and NEMO [81]. All of these functions of A20 inhibit downstream NFκB signalling.

The deubiquitinase CYLD removes K63-polyubiquitin chains from RIP1, TRAF2, TRAF6, TAK1, and NEMO, as well as removing M1-linear polyubiquitination from RIP1 [23]. As such, CYLD functions as a tumor suppressor gene in several cancers [75]. Other deubiquitinases can also modulate NFκB signalling. USP4, USP20, and USP21 can remove K63-polyubiquitin chains from RIP1 (USP4 and USP21) and TRAF6 (USP20), respectively [82,83,84]. Furthermore, USP4 can remove K63-polyubiquitin chains from TAK1, whereas USP21 can remove K63-polyubiquitin chains from TRAFConversely, the OTU family member OTUB1 can stabilize both cIAP1/2 and UBC13, resulting in enhanced NFκB signalling [85]. Additionally, USP11 and USP15 can deubiquitinate and stabilize IκBα, promoting NFκB nuclear translocation [86].

The deubiquitinases OTUD7b, USP48, and USP7 can regulate NFκB signalling at multiple levels. OTUD7b can remove K11- and K63-polyubiquitin chains from RIP1 [87]. In contrast, USP7 can remove K48-polyubiquitin chains from IKKα and p65, thereby promoting NFκB signalling [88], and USP48 stabilizes TRAF2 and p65 by the removal of K48- polyubiquitin chains [89,90].

#### 2.3.2. Non-Canonical NFκB Signalling

In contrast to canonical NFκB signalling, the regulation of non-canonical NFκB signalling is not as well understood (Figure 3). In unstimulated cells, NIK is constantly turned-over by *TRAF3*, which, in combination with another E3 ligase C terminus of HSC70-Interacting Protein (CHIP), promotes the K48-polyubiquitination and proteasomal degradation of NIK [38,91]. Upon ligand activation, cIAP-mediated K48-polyubiquitination of TRAF3 promotes its proteasomal degradation; this induces NIK accumulation and the subsequent activation of IKKα [38]. Interestingly, the E3 ligase ZFP91 can stabilize and activate NIK [92]. Downstream, SCF^β-TrCP^ can induce K48-polyubiquitination of both p100 and p105, whereas SCF^FBXW7α^ can induce K48-polyubiquitination of p100, targeting them for partial proteasomal degradation [29]. This liberates p52 and p50, inducing their nuclear translocation and subsequent NFκB activation.

The only deubiquitinases identified to play a role in non-canonical NFκB signalling are OTUD7b and OTUBOTUD7b and OTUB1 remove K48-polyubiquitin chains from TRAF3 and cIAP1, respectively [85,87]; therefore, OTUD7b and OTUB1 can inhibit both the canonical and non-canonical NFκB pathways.

It is thus evident that the modulation of NFκB signalling by the ubiquitin system is complex, with many E3 ligases and DUBs targeting multiple components to ensure efficient regulation. This suggests that defects in the ubiquitin system can result in chronic NFκB signalling and promote cancer progression.

## 3. Ubiquitin-Mediated NFκB Signalling in Head and Neck Squamous Cell Carcinoma

### NFκB Signalling in HNSCC

NFκB signalling drives cell proliferation, survival, therapeutic resistance, and pro-inflammatory factors [93,94]. As mentioned previously, NFκB is often aberrantly activated in HNSCC, including oropharyngeal, laryngeal, hypopharyngeal, and tongue cancers [95,96,97,98]. Additionally, NFκB signalling plays an important role in other cancers of the head and neck distinct from HNSCC, such as salivary gland cancer and nasopharyngeal carcinoma [99,100,101]. Early studies from our lab demonstrated that key components of the canonical NFκB pathway are aberrantly activated in HNSCC and are further activated by TNFα via the IKK complex [22,102,103]. Furthermore, recent studies have demonstrated the activation of non-canonical NFκB signalling, involving NIK and IKKα [104,105,106]. Both of the NFκB pathways have also been shown to interact with other pro-oncogenic signalling pathways, including the EGFR, STAT3, and AP-1 pathways [104,107,108].

The activation of NFκB in HNSCC occurs through various mechanisms, including somatic mutations, genetic copy number alterations (CNA), or the expression of the HPV viral oncogenes [14,19,20]. Several genome wide studies have highlighted the prevalence of several alterations in the ubiquitin system implicated in the aberrant activation of NFκB signalling in HNSCC (Table 2).

In the TCGA HNSCC dataset, frequent amplification and overexpression of *BIRC2/3* (on chromosome 11q22), encoding cIAP1/2, was observed [109]. These E3 ligases are the critical mediator of TNFα signalling, promoting K63-polyubiquitination of RIP1 and K48-polyubiquitination and subsequent proteasomal degradation of TRAF3 [36]. *BIRC2* is more commonly amplified and overexpressed in HPV- HNSCC, while BIRC3 overexpression appears to be more common in HPV+ HNSCC [109]. Additional ubiquitin components are upregulated at the mRNA level; OTUB1 mRNA levels are high in HNSCC, without significant copy number alterations [14].

A key finding of the TCGA study was the identification of common mutations/deletions in the *CYLD* and *TRAF3* genes in HPV+ HNSCC [17,110]. CYLD removes both K63-polyubiquitin and M1 linear-ubiquitin chains, inhibiting NFκB signalling at several different steps [111]. TRAF3 is a negative regulator of both classical and alternate NFκB [38,112]. Further studies demonstrated that these mutations in *CYLD/TRAF3,* or the loss of *TRAF3*, resulted in enhanced NFκB signalling and promoted HPV+ HNSCC [105,110]. Intriguingly, loss of *CYLD/TRAF3* was observed in a subset of HPV+ HNSCC with episomal HPV, suggesting loss may enhance the establishment of persistent infection and susceptibility to transformation [110]. Furthermore, similar genomic alterations in *CYLD* and/or *TRAF3* were observed salivary cancers and in a cohort of nasopharyngeal carcinomas negative for the Epstein Barr Virus (EBV) [101,113,114].

The mutational analysis also demonstrated that SCF^FBXW7^ is one of the most commonly mutated genes in HPV− HNSCC [17]; this E3 ligase promotes the degradation of the NFκB protein p100, inhibiting NFκB activity [112]. FBXW7 ‘hot-spot’ mutations (R505 > R479 > R465) occur in the substrate binding domain; these mutations inhibit the interaction between SCF^FBXW7^ and its substrates [115]. Therefore, these mutations may promote the stabilization of p100, potentially increasing the inactive precursor for stimuli-induced processing, thereby enhancing NFκB activity [116].

Cullin ring ligase 3 (CUL3) is an E3 ligase, and KEAP1 is a CUL3-specific adapter protein. The most well studied substrate inhibited by the CUL3-KEAP1 complex is Nuclear factor erythroid 2-related factor 2 (NRF2), a transcription factor that regulates antioxidant and stress-responsive genes [117]. Co-activated NRF2 and NF-kB may cooperate in transcriptional activation [117]. The CUL3-KEAP1 complex also targets IKKβ for proteasomal degradation, inhibiting NFκB signalling [67]. Thus, mutations, or loss, of either *CUL3* or *KEAP1* can result in increased inflammatory signalling through co-activation of NFκB and NRFAdditionally, in non-tumorigenic Retinal Pigment Epithelium RPE cells, *CUL3* inactivation in combination with *TTP53* inactivation results in an oncogenic phenotype driven by NFκB and AP-1 signalling [118]. In line with this, mutations in *CUL3* significantly co-occur with mutations in *TTP53* in HNSCC [119], suggesting that the CUL3-KEAP1 E3 ligase complex may function in concert with deleted, mutated, or degraded *TTP53* in HNSCC to promote NFκB, AP-1, and NRF2 activation.

These differences in genomic alterations in HPV+ and HPV− HNSCC demonstrate that the activation of NFκB signalling can occur by different mechanisms. However, HPV itself can also induce NFκB activation; HPV E6 has been shown to promote NFκB activation by several mechanisms [19,20]. Interestingly, E6 mediates the proteasomal degradation CYLD in hypoxic conditions [20], an environment that occurs in the poorly vascularized center of cervical lymph nodes common in HPV+ HNSCC. Recently, it has been shown that E6 can also activate the GTPase Rac1, which regulates NFκB activity in cervical cancer cells [19]. Thus, it is clear that HPV can induce NFκB activity by a number of mechanisms that could potentially be specifically targeted for therapy for HPV+ HNSCC.

## 4. Targeting the Ubiquitin System to Inhibit NFκB Signalling in HNSCC

The success of kinase inhibitors in cancer treatment over the past 30 years, along with recent research into how defects in the ubiquitin system contribute to disease, has prompted the pharmaceutical industry to investigate the ubiquitin system as a potential therapeutic target [120]. So far, progress has been slow, and only a few small molecule inhibitors have been successfully developed and are clinically approved or in clinical trials. This may be due to the complex and dynamic nature of ubiquitination, which involves many protein-protein interactions that have been challenging to disrupt by small molecules with the necessary specificity to avoid toxicity.

In spite of this, recent advances in the understanding of ubiquitination biology have identified new small molecule inhibitors targeting components of the ubiquitin system that are efficacious in several diseases, including cancer [120]. Below, we will review how targeting various components of the ubiquitin system could be utilized to regulate the NFκB pathway in HNSCC.

### 4.1. Targeting the Proteasome

The most advanced inhibitors of the ubiquitin system are proteasome inhibitors. The inhibition of the proteasome deregulates protein turnover, resulting in the stabilization of tumor suppressors, such as TP53 [121]. Additionally, proteasome inhibition can inhibit the NFκB pathway by allowing the accumulation of phosphorylated IκBα [22]. Thus, proteasome inhibition can have multiple effects that inhibit cell proliferation and promote apoptosis.

Since the mid 1990s, proteasome inhibitors have been tested in many cancers, including multiple myeloma (MM) and various lymphomas [121]. Currently, two proteasome inhibitors are approved by the FDA, bortezomib (Velcade^®^, Millenium Pharmaceuticals, Cambridge, MA, USA) and carfilzomib (Kyprolis^®^, Amgen, Thousand Oaks, CA, USA) [122]. Bortezomib is a first-generation proteasome inhibitor, which reversibly binds to the catalytic site of the proteasome [123]. Bortezomib is currently FDA approved for the treatment of MM and mantle-cell lymphoma (MCL). Studies have shown that bortezomib’s efficacy in MM and MCL is likely due to multiple mechanisms, including the inhibition of proliferation and the induction of apoptosis [124,125]. Additionally, bortezomib inhibits NFκB signalling due to the stabilization of IκBα [126]. Unfortunately, bortezomib treatment often results in intrinsic or acquired resistance in many patients [123]. Therefore, the second-generation of proteasome inhibitors were designed to overcome this; carfilzomib is an irreversible inhibitor of the proteasome that is currently approved for the treatment of MM that is refractory to at least two previous therapeutics, including bortezomib [127].

Proteasome inhibitors have been tested as a potential therapeutic option in HNSCC. In early clinical studies, bortezomib treatment resulted in clinical tumor shrinkage or necrosis in a subset of patients [22,128]. In both mouse and human squamous cell carcinomas, bortezomib treatment was shown to inhibit NFκB signalling [22,129]. Furthermore, it inhibited canonical NFκB signalling to a greater extent than non-canonical NFκB signalling [18]. When combined with anti-EGFR antibody cetuximab and radiation, these effects were found to be negated by the inhibitory effect of bortezomib on the degradation of EGFR, which can promote resistance via activation of NFκB and MAPK-AP-1 axes [130]. As a result of early recurrences observed, this phase I clinical trial of bortezomib in combination with chemoradiotherapy and cetuximab in stage IV HNSCC was halted (Table 3). Additional pre-clinical studies demonstrated that when combined with histone deacetylase (HDAC) inhibitors or cisplatin, bortezomib enhanced the induction of apoptosis and therapeutic responses [131]. However, these combinations were relatively toxic and offered too narrow a therapeutic window in preclinical models.

### 4.2. Targeting E1 Activating Enzymes

Ubiquitin activating enzymes (UBEs or E1 enzymes) are the first step of the ubiquitination cascade (Figure 1). E1 enzymes catalyze the formation of a thioester bond between the C-terminal carboxyl group of ubiquitin and the cysteine residue of E1 in an ATP-dependent manner [120]. To date, eight E1 enzymes have been identified in humans that regulate the addition of ubiquitin and ubiquitin-like proteins (UBLs) to protein substrates. Only two E1 enzymes, UBA1 and UBA6, control the ubiquitination of all proteins.

PYR-41 and PYZD-4409 were the earliest identified inhibitors for UBA1; both inhibitors irreversibly inhibit UBA1 by covalent attachment to the active cysteine (Cys^632^) residue [134,135]. Both inhibitors have demonstrated some anti-tumor effects in vitro in leukemia cell lines and can inhibit NFκB signalling. However, the apoptosis observed is likely due to off target effects caused by the global inhibition of ubiquitination [134]. A more potent inhibitor of UBA1 called MLN7243 (also known as TAK-243) forms a covalent adduct with ubiquitin and, in contrast to PYR-41 and PYZD-4409, inhibits UBA6 as well as UBAMLN7243 has shown anti-tumor activity in vivo in immunodeficient mice subcutaneously grafted primary human AML cells [136]. However, in a phase I dose-escalation clinical trial (clinicaltrial.gov identifier: NCT02045095) in advanced solid tumors, over one third of patients had serious adverse effects, suggesting that such global inhibition of ubiquitination is toxic.

In addition to the two E1 activating enzymes involved in the ubiquitination pathway, the E1 activating enzyme UBA3 should be mentioned. UBA3, also known as the neuronal precursor cell, expressed, developmentally down-regulated 8 (NEDD8)-activating enzyme (NAE)2, is the E1 activating enzyme of the ubiquitin-like protein NEDD8 [137]. This E1 functions in concert with an additional subunit, NAE1; the heterodimer of NAE1 and 2 is normally just referred as NAE.

The addition of NEDD8 to protein substrates is termed NEDDylation. Whilst this pathway is not part of the ubiquitin system, NEDDylation is essential for the activation and function of the Cullin Ring Ligase (CRL) family of E3 ligases [138]. The cullin subunit of CRLs is NEDDylated, and CRL E3 ligase is often involved in cell proliferation. Several are deregulated in cancer, including HNSCC (17); thus, inhibition of CRL E3 ligases results in the accumulation of CRL substrates.

MLN4924 (Pevonedistat) is an NAE inhibitor that forms a covalent adduct with NEDD8 and is currently in Phase I/II trials for various types of leukemia (clinicaltrial.gov identifier: NCT03268954). Initial experiments showed that MLN4924 induced DNA damage in vitro and limited tumor growth when used to treat immunocompromised mice in vivo [139].

Several studies have shown that MLN4924 inhibits NFκB signalling; in both chronic lymphocytic leukemia (CLL) and non-Hodgkin lymphoma, MLN4924 stabilized phosphorylated IκBα, inhibiting p65 and p50 nuclear translocation, NFκB activity and inducing apoptosis [139]. In HNSCC, MLN4924 has anti-tumor effects, inducing apoptosis and sensitizing HNSCC cells to radiation and TRAIL-induced cell death [140,141,142]. However, the anti-tumor mechanism of MLN4924 in HNSCC is still unclear, and no clinical trials in HNSCC have been performed.

### 4.3. Targeting E2 Conjugating Enzymes

The E2 ubiquitin-conjugating enzymes interact with numerous E3 ligases to transfer ubiquitin molecules onto substrate proteins (Figure 1). Over 30 identified E2 genes in humans [143]; therefore, targeting E2 enzymes should provide more selectivity than E1 enzymes. However, only a few inhibitors targeting E2 conjugating enzymes have been discovered so far, with none currently in clinical trials.

NSC697923 is an irreversible, covalent inhibitor of the UBC13/UBE2V1 heterodimer that catalyzes the formation of K63-polyubiquitin chains [144,145]. Inhibition occurs by covalent attachment of NSC697923 to the catalytic cysteine of UBC13, preventing the conjugation of ubiquitin [145]. Interestingly, NSC697923 was originally identified in a screen for molecules inhibiting the NFκB pathway [144]. The research demonstrated that NSC697923 suppressed the proliferation of diffuse large B-cell lymphoma (DLBCL) cells in vitro, via the inhibition of the NFκB pathway [144]. Another UBC13 inhibitor, BAY117082, was first thought to inhibit the IKK complex, as it inhibited IκBα phosphorylation [146]; however, BAY117082 covalently attaches to the catalytic cysteine of UBC13 and UBCH7 [145]. This results in the inhibition of both K63-polyubiquitin and M1 linear-polyubiquitin chain formation in lymphoma cells, promoting cell death.

### 4.4. Targeting E3 Ligases

As the largest enzyme class involved in the ubiquitination cascade, over 700 E3 ligases are currently identified [41]. These are subdivided into at least three sub-classes, based on structural classification: the Really Interesting New Gene (RING) E3s, which function as a scaffold to bring ubiquitin-conjugated E2 enzymes in close contact with their substrates; Homologous to E6-AP Carboxyl Terminus (HECT) E3s, which catalyze the transfer of ubiquitin to their own cysteine residues before transfer to substrates, and a third subfamily, RING-Between-RING (RBR) E3s, which function as a hybrid between RING and HECT E3s [41].

As a large family of enzymes that use distinct catalytic mechanisms, the targeting of E3 ligases should offer a high degree of specificity, resulting in less toxicity and having greater therapeutic potential. Many E3 ligase inhibitors have been identified; here, we will focus on E3 ligases, which have been implicated in NFκB signalling.

As mention previously, MLN4924 can impact the function of the CRL sub-family of E3 ligases. CRLs play a critical role in NFκB activation [147]. The SCF E3 ligase complex containing CUL1 is the most well characterized to date. SCF*^β^*^-TrCP^ induces K48-polyubiquitination and the subsequent proteasomal degradation of IκBα, as well as the partial cleavage of the NFκB proteins p100 and p105 [34,148]. Furthermore, p100 is also a target for SCF^FBXW7α^ [116]. The KEAP1-Cul3-RBX1 CRL E3 ligase complex, containing CUL3, induces IKKβ K48-polyubiquitination and proteasomal degradation [67]. MLN4924 inhibits the function of all cullin proteins; thus, inhibition of NAE could inhibit proliferation and induce cell death in HNSCC by inhibiting the NFκB pathway.

Of particular interest, the *KEAP1,* and *CUL3* genes are among the most frequently mutated proteins in HNSCC [17,119]. Therefore, loss of function mutations in these three genes may contribute to the constitutive activation of NFκB in HNSCC and in other cancers [118]. This suggests that the inhibition of cullin activity via MLN4924 may function similarly to loss of function mutations observed in these genes in HNSCC patients and may have the potential to inhibit the NFκB pathway in these cancers.

The dual function of cIAP1 and 2 has made them an interesting potential therapeutic target in cancer by inhibiting NFκB and downstream inflammatory signalling and the induction of apoptosis though the extrinsic pathway [149]. Compounds targeting cIAPs, so called IAP antagonists, are mimicked after the natural IAP antagonist, Second Mitochondria-derived Activator of Caspases (SMAC)—thus, they are also called SMAC mimetics. Upon mitochondrial release, SMAC is cleaved and dimerizes. The N-terminal domain of SMAC contains a four amino acid sequence (AVPI; alanine (A), valine (V), proline (P), isoleucine (I)); this sequence binds to the BIR3 and BIR2 domain of IAPs [149]. IAP antagonists mimic this sequence and bind to IAPs. Upon binding, cIAP1/2 undergo a conformation change, autoubiquitination, and proteasomal degradation. In addition, some SMAC mimetics target XIAP, preventing it from binding to caspase 3, 7, and 9, thereby inducing caspase activation and apoptotic cell death [150]. The IAP antagonists developed thus far are monovalent or bivalent, based on how many BIR domains are bound by the IAP antagonist. The mechanism of action IAP antagonists is primarily the enhancement of TNFα-dependent apoptosis rather than inhibition of NFκB; despite the potential for canonical NFκB signalling inhibition, cIAP depletion results in stabilization of NIK, thereby activating non-canonical NFκB signalling [149]. This results in autocrine TNFα-signalling by tumor cells, as well as enhancing TNF*α* production in cytotoxic T lymphocytes. Altogether, increased TNF*α* in the microenvironment and the induction of caspases in tumor cells favors the induction of apoptotic and necroptotic cell death. IAP antagonist-induced apoptosis is also enhanced in combination with other death ligands, such as TRAIL [151].

A number of IAP antagonists are currently in clinical trials; four monovalent (Debio-1143, GDC-0917, LCL-161, and GDC-0152), two bivalent (birinapant and HGS-1029), and a synthetic based on the binding pocket rather than an AVPI peptidomimetic (ASTX-660). In HNSCC, several IAP antagonists have been shown to have activity in vitro. Birinapant and Smac-164 sensitize HNSCC cancer cells to standard chemotherapeutics, TNFα and TRAIL [152]. In addition, Debio-1143 and LCL161 sensitize HNSCC cells to radiation therapy [64,65]. Interestingly, HNSCC cell lines with amplification of the Fas-associated death domain (*FADD)*, which mediates TNFR induced cell death, had an increased sensitivity to IAP antagonists and combination therapy with radiation, and resulted in an enhanced expression of TNFα [109]. Furthermore, IAP antagonists enhanced sensitivity to death ligands in HPV+ HNSCC that lack *FADD* copy gains. In some of these lines, IAP antagonists enhanced TP53 expression, which is targeted for degradation by HPV EIn these studies, ASTX660, in combination with TNFα and TRAIL, induced cell death in HNSCC cell lines and radiation-induced immunogenic death in pre-clinical models [151,153]. ASTX660 is currently in phase I trials in advanced solid tumors and lymphomas (Table 3; NCT02503423). Recently, Debio-1143 was granted breakthrough therapy designation by the Food and Drug Administration (FDA) to treat untreated, unresectable, and locally advanced HNSCC in combination with standard chemoradiation therapy. The phase II study results (Table 3; NCT02022098) presented at the ESMO Congress 2019 in Barcelona, Spain revealed a significant improvement for the primary endpoint locoregional control rate, 18 months after CRT (21% improvement vs. control arm) and a progression-free survival (PFS) benefit vs. the CRT + placebo arm after a 2-year follow-up period (HR = 0.37, *p* = 0.007). The drug showed a manageable safety profile, and that it did not compromise the full delivery of standard CRT. Therefore, these data suggest that IAP antagonists offer clinical benefit in HNSCC patients, particularly when the underlying genetics or HPV status of a tumor are known, making them an attractive target in personalized medicine. Currently, Debio 1143 is in phase I/II trials in combination with nivolumab, an anti-PD-1 monoclonal antibody approved for treating a number of cancers, including HNSCC (Table 3; NCT04122625).

### 4.5. Targeting Deubiquitinase

Like E3 ligases, several deubiquitinases are deregulated and implicated in cancer development [154]. Due to their well-defined active site, deubiquitinases are currently gaining much interest as potential drug targets. Among all known deubiquitinases, USP7 inhibitors are the most advanced due to its critical role in regulating TP53 function by deubiquitinating the TP53 negative regulator MDM2 [155]. Furthermore, USP7 directly deubiquitinates RELA/p65, promoting NFκB signalling [156]. The early USP7 inhibitors P5091 and HBX19818 were modestly efficacious, fairly selective, and induced TP53-dependent cell death in vivo [157]. Importantly, P5091 induced apoptosis in MM patient cells, including those resistant to prior treatments such as bortezomib [157]. More recently, highly potent and specific USP7 inhibitors demonstrated high efficacy in vitro and MM xenografts in mice [158]. Despite these positive studies, USP7 inhibitors may only stabilize wild-type TP53, whereas the majority of tumors harbor mutant TP53; therefore, the clinical efficacy of these inhibitors will depend on the genetic background of the tumor.

The removal of ubiquitin chains from target proteins during proteasomal degradation is performed by proteasome-associated deubiquitinases, including UCHL5 and USP14 [159]. These deubiquitinases trim polyubiquitin chains to allow substrate proteins entry into the proteasome for degradation. The inhibitor b-AP15 targets both UCHL5 and USP14 and leads to the accumulation of ubiquitinated substrates [160]. Furthermore, due to its effects on proteasomal degradation, b-AP15 can result in the inhibition of NFκB signalling [161]. b-AP15 demonstrated exhibited excellent efficacy in different in vivo solid tumor models [160]. VLX1570, a b-AP15 analogue with higher potency and higher selectivity for USP14, demonstrated anti-tumor activity, and enhanced survival in MM xenografts [162]. However, a recently reported Phase 1 study of VLX1570 in multiple myeloma patients showed that despite some preliminary anti-tumor effects, treatment resulted in severe toxicity in several patients, similar to previous observations after treatment with proteasome inhibitors [163]. USP14 has also been shown to regulate NFκB via the deubiquitination of IκBα [164]. More selective USP14 inhibitors are now available; IU1, which inhibits USP14 but not UCHL5, led to tumor regression in a TP53-deficient mouse tumor model [165].

## 5. Conclusions

Many of the ubiquitin activating and proteasomal degradation steps hold great potential as drug targets. However, they also suffer from issues of low specificity. In contrast, advances in computational chemistry and novel technologies, including mass spectrometry and high-throughput screening, the development and success of specific E3 ligase and deubiquitinase inhibitors with more selective anti-cancer activity may be possible. These new technologies may enable the development of innovative therapeutic approaches that target protein-protein interactions, or co-opt the ubiquitin system, as is the case with protein-targeting chimeric molecules, which bind to protein and induce their proteasomal degradation (PROTACs) [166]. It is clear that the NFκB pathway is essential for the proliferation and survival of HNSCC, in vitro and in vivo. Furthermore, targeting the ubiquitin system is an attractive therapeutic target in HNSCC due to the potential to target the NFκB pathway. The use of proteasome inhibitors has demonstrated that this strategy has clinical benefit in multiple myeloma, but toxicity, lack of specificity, and resistance mechanisms emerged as a challenge in HNSCC. Thus, further research into the role of specific enzymes of the ubiquitin system and how they regulate the NFκB pathway may offer novel therapeutic strategies in HNSCC.

## Figures and Tables

**Table 1:** Overview of the key differences between HPV− and HPV+ HNSCC.

Features	HPV− HNSCC	HPV+ HNSCC
**Clinical and epidemiological characteristics**		
**Incidence**	Decreasing	Increasing
**Age**	Detected mostly in older adults	Detected mostly in young adults
**Tumor Location**	All parts of the head and neck	Oropharynx, especially the tonsil and base of the tongue
**Prognosis**	Poor	Good
**Response to Therapy**	Unfavorable response to chemoradiation	Favorable response to chemoradiation
**Survival**	Low survival rate	High survival rate
**Biological and histological characteristics**		
**TP53 Status**	Highly mutated	Degraded by HPV E6
**p16^INK4a^ Status**	Decreased expression, due to inactivating mutations, deletions or methylation	Frequently overexpressed
**Genomic Stability**	Genome is highly unstable	Genome is less unstable
**Regional Metastasis**	Frequent. Less bulky	More frequent, bulky
**Distant Metastasis**	Frequent	Rare
**Secondary malignancy**	Frequent	Rare
**Histology**	Moderately or well differentiated squamous cell carcinoma; keratinized	Poorly differentiated squamous cell carcinoma; basaloid

**Table 2:** Overview of ubiquitin-related genes modulated in HNSCC and have a potential role in NFκB activity.

Gene	Genomic Status	Known Role in NFκB in HNSCC?
***BIRC2/3***	Amplification, mRNA upregulation	√
***TRAF3***	Deletion, mutation	√
***OTUB1***	mRNA upregulation	X
***CYLD***	Mutation	√
***FBXW7*** *α*	Mutation	X
***CUL3***	Mutation	X
***KEAP1***	Mutation	X

**Table 3:** Overview of clinical trials for ubiquitin-modulating compound for HNSCC.

Compound	Co-Therapy	Phase	Clinical Trial Number or Reference
**Bortezomib**	Cetuximab and radiation +/− cisplatin	I	NCT01445405
**Bortezomib**	Reirradiation	I	[132]
**Bortezomib**	Docetaxel	II	[128]
**Bortezomib**	Radiation + cisplatin	I	[133]
**Debio 1143**	Radiation + cisplatin	I/II	NCT02022098
**Debio 1143**	Nivolumab	I	NCT04122625
**ASTX660**	N/A	I	NCT02503423
**Birinapant**	Radiation	I	NCT03803774
